# Composites from Recycled Polypropylene and Carboxymethylcellulose with Potential Uses in the Interior Design of Vehicles

**DOI:** 10.3390/polym16152188

**Published:** 2024-07-31

**Authors:** Alina Ruxandra Caramitu, Romeo Cristian Ciobanu, Ioana Ion, Mihai Marin, Eduard-Marius Lungulescu, Virgil Marinescu, Magdalena Aflori, Adriana Mariana Bors

**Affiliations:** 1 National Institute for Research and Development in Electrical Engineering—ICPE-CA, 030138 Bucharest, Romania; alina.caramitu@icpe-ca.ro (A.R.C.); ioana.ion@icpe-ca.ro (I.I.); mihai.marin@icpe-ca.ro (M.M.); marius.lungulescu@icpe-ca.ro (E.-M.L.); virgil.marinescu@icpe-ca.ro (V.M.); 2 Department of Electrical Measurements and Materials, Gheorghe Asachi Technical University, 700050 Iasi, Romania; 3 Petru Poni Institute of Macromolecular Chemistry, 41 A Gr. Ghica Voda Alley, 700487 Iasi, Romania; maflori@icmpp.ro; 4 National Research and Development Institute for Optoelectronics-INOE 2000-IHP, 040558 Bucharest, Romania; bors.ihp@fluidas.ro

**Keywords:** recycled polypropylene, cellulose, polymer composite

## Abstract

This research investigates novel polymeric composite materials for automotive interior trim applications. The composites utilize recycled polypropylene (PPr) matrix and carboxymethylcellulose (CMC) as filler (PPr/CMC: 100/0, 95/5, and 90/10 wt.%). The materials were processed by extrusion and injection molding. Considering their intended application, the composites were evaluated for resistance to key climatic factors, i.e., temperature, humidity, and UV radiation. In addition, structural analyses and FTIR analyses were performed to assess potential heterogeneity and thermal stability. Following FTIR tests, the incorporation of carboxymethyl cellulose in polypropylene is confirmed by the detection of characteristic CMC bands for -OH, C=O, and C-O-C groups. The results indicate slight structural heterogeneity in the 5% and 10% CMC composites. However, no thermal distortions were observed in either the composites or the PPr matrix itself. The behavior of PPr/CMC composites under the action of the mentioned climatic factors has been assessed from the variation of dielectric characteristics with frequency. The strong polarization of CMC leads to a sharp increase in composites electrical conductivity after submersion in water for 480 h, suggesting weakening of the composite structure. After exposure to UV radiation, a sharp increase in conductivity is observed even after the first cycle (72 h) of UV radiation. Following the experimental results obtained in our study, it is recommended to use the PPr +10% CMC composite for obtaining different interior ornaments (carpets, supports, etc.). At the same time, the use of these materials also has the advantage of lightening the mass of the vehicle due to their lower density than polymers.

## 1. Introduction

Plastic production and waste are increasing nationally and internationally. Plastic pollution causes major problems in the world because it poses health and environmental hazards [1,2]. Plastic recycling is a circular economy that can turn this plastic waste into resources while reducing pollution and helping to achieve the Sustainable Development Goals (SDGs). Thermoplastics, which account for around 80% of all plastics used in consumer goods and are mainly used as packaging or textile fibers, can be effectively recycled to reduce landfill waste and demand for new raw materials [3]. Polypropylene (PP), high- and low-density polyethylene (HDPE and LDPE), polyvinyl chloride (PVC), polystyrene (PS), and others are some of the most important thermoplastic polymers [4,5]. Hence, recycling and reusing plastic will not only help clean up the environment but also allow the government to generate a lot of money by recycling and exporting plastic [6]. In fact, recycling effectively contributes to reducing production costs [7]. Companies such as THRIVE produce a variety of injection-molded composites from virgin and recycled polypropylene (PPr) and cellulose for use in automotive, home appliances, furniture, building materials, sports and leisure, and personal and household goods [5]. The study of the dielectric characteristics of cellulose began in 1938 with the measurement of the dielectric constant of the cellulose suspension [8], so in 1977 [9], the dielectric constants of different cellulose were comparatively studied, and it was found that the dielectric properties of cellulose depend mainly on the presence of hydroxyl groups.

In cellulose, the dielectric constant, as an indicator of polarization, plays a major role related to the transfer of electrons, ions, and direction polarization. Other studies indicate that, like many polymeric materials, cellulose has a relatively low dielectric constant ε (3.9 to 7.5) and dielectric losses less than ~0.1 [10,11]. In general, the more polar the polymer, the higher the dielectric constant presents.

The dielectric properties of cellulose are affected by many factors, such as its intrinsic properties (types and number of polar groups, hydrogen bonds, crystallinity degree and crystallite size, degree of polymerization, density, cellulose source, hemicellulose and lignin content, preparation and purification method, impurities, etc.) and environmental factors (ambient temperature and humidity, UV radiation intensity and wave lengths, etc.) [12,13,14].

The reinforcement of a polypropylene polymer matrix with CMC creates new dielectric characteristics and induces superior properties (thermal, mechanical, chemical, etc.) for the obtained composite compared to the characteristics of each component taken separately [15,16]. Dielectric composites prepared by mixing CMC with polymers are less studied. This limitation is due to the lack of information about their structure and dielectric behavior. Unlike highly dielectric polymer matrixes, natural compounds have obvious advantages in flexibility and degradability and have great potential in the field of highly dielectric materials, which can be widely used in energy, environment, healthcare, and other industries [17]. *Anju and Narayanankutty* [18] in 2016 proposed a new nanocomposite system, which used PVA and polyaniline (PANI) to coat nanocellulose. The main properties of the most well-known electrical insulators, conventional polymers, such as HDPE (high-density polyethylene), LDPE (low-density polyethylene), PP (polypropylene), PVC (polyvinyl chloride), etc. were presented in [19,20,21,22,23,24,25,26].

Polymer-based dielectrics have proven to be the most attractive materials, mainly due to their excellent mechanical and dielectric properties, good chemical resistance, ease of obtainability, and low production costs [27,28]. Moreover, the application of such materials is important for the insulation of electrical cables, for a reliable power transmission network, and for a better insulation system between conductive parts. Indeed, polymers and composites with a polymer matrix are well suited for energy storage functions in electrical engineering, as in many other fields (aeronautics, automotive, construction, etc.). Several studies have shown improvements in mechanical and physical properties made to thermoplastic polymers by adding cellulose fibers [29,30,31]. Other studies have presented the dielectric properties of biodegradable composites based on the addition of natural fibers (jute, sisal, hemp, flax, etc.) or wood as a filler in a polymer matrix [32,33].

At normal temperatures, brittle fracture of thermoplastic composites can already be observed at filling ratios of 10–15% by volume. The addition of rigid filler particles produces an increase in the modulus of elasticity proportional to the volume percentage of the filler. The effective surface energy of the fracture is higher in the composite than in the unfilled polymer because the dispersed particles make the crack propagation path longer, absorb part of the energy, and increase the plastic deformation of the matrix. It becomes evident that the larger the size of the particles of the filler material, the larger will be the voids formed. The void content is high in many composites due to poor wetting of the filler particles by the polymer. At the same time, the content of voids can be associated with areas of agglomeration of the particles of the dispersed filler material, a phenomenon that is reflected in the decrease in the mechanical strength of the composite due to the increase in the size of the particles of the filler material and the low strength of the agglomerates themselves. These agglomerations can be reduced by using certain coupling agents [34]. Agglomerations of fibers/filling particles from composite materials can cause cracks in the polymer matrix, these becoming nuclei from where the splitting of the polymer chains can begin and implicitly its grinding. In order to obtain certain biodegradable materials in the natural environment, it is necessary to add high concentrations of natural fibers (wood cellulose, flax, etc.) to the polymer structure (greater than 50%), but these concentrations seriously affect the mechanical characteristics of the composite; therefore, we tried small percentages of natural fibers (5 and 10%, respectively) to create, through agglomerations, centers in the mass of the polymer where degradation can begin [34,35].

The parts of the car that could be made of composite materials are door interiors, the rear shelf above the luggage compartment, the center console, different silencers and different insulation, pillars, and seat faces.

Composite materials present different states of dispersion of filler particles (cellulose) that form groups of cells in the polymer matrix depending on their volume, diameter, and distribution [28,29,30]. *Elloumi* et al. [35] in 2021 reported a close relationship between the cellulose content of the composite and the dielectric constant. Thus, cellulose-based biocomposites would be superior from a dielectric point of view to biocomposites made of wood or natural fibers. However, only a few studies [36,37,38,39] have investigated the impact of cellulose fibers on the dielectric properties of WPCs in the low frequency range (1 Hz–1 MHz).

The purpose and, at the same time, the novelty of this research is to obtain new composite materials of the PP/CMC type from recycled sources and, at the same time, to identify (through dielectric tests) the composite that degrades the fastest under the action of environmental factors (temperature, UV radiation, and moisture). These materials will find their use in making different profiles in the automotive industry because they have a lower density than the plastic material without filling, which leads to a decrease in the total weight of the vehicle, a reduction in fuel consumption, and at the same time, a decrease in emissions from the exhaust to the atmosphere.

## 2. Materials and Methods

### 2.1. Materials

The raw materials used to obtain the polymer composite materials are as follows: (1) Matrix: recycled polypropylene (PPr) from electronic waste [39]. The use of recycled polypropylene is in line with Directive 2000/53/EU on end-of-life vehicles. (2) Filler: carboxymethyl cellulose medium viscosity (Sigma Aldrich, Saint Luis, MO, USA) [40]

### 2.2. Methods

#### 2.2.1. Obtain Methods

**Figure 1:**
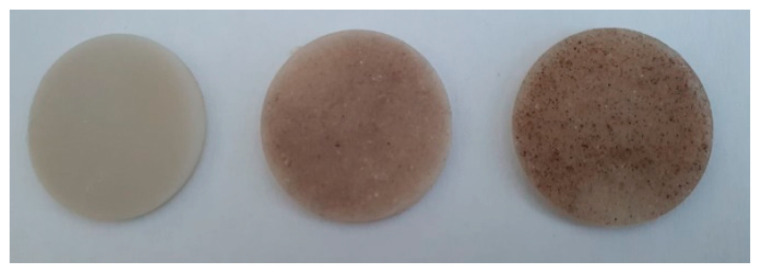
PPr/CMC composite material samples for dielectric tests.

The polymer composite materials type PP/CMC were obtained in two stages, i.e., composite pellets by extrusion, by use of a laboratory twin screw extruder Lab-Compounder KETSE 20/40 (Brabender, Duisburg, Germany), followed by a melt injection process with a Dr. Boy 35A injection-molding machine (Germany) in order to obtain testing samples. As regards the injection process, the working temperatures of 170–230 °C and clamping forces of 138–155 kN were used to achieve disk-shaped materials with a diameter of 30 ± 0.1 mm and a thickness of 2 ± 0.1 mm. To obtain the PPrr/CMC type composites, 3 concentrations of CMC were used, and the performances obtained for each were compared in order to select an optimal variant for the proposed use. Thus, the concentrations and coding of the 3 types of composites were presented in Figure 1 and Table 1. Regarding the extrusion process, temperatures were used on the areas of the extruder, in the range of 190–240 °C, and in the injection process, working temperatures of 170–230 °C (Table 1) and forces of 138–155 kN were used to make disk-shaped materials with a diameter of 30 ± 0.1 mm and a thickness of 2 ± 0.1 mm. To obtain the PPr/CMC type composites, 3 concentrations of CMC were used, and the performances obtained for each were compared in order to select an optimal variant for the proposed use. Thus, the concentrations and coding of the 3 types of composites were presented in Figure 1 and Table 2.

#### 2.2.2. Characterization Methods

##### Structural Analysis—SEM (Scanning Electron Microscopy) Analysis

Scanning electron microscope with field emission source and focused ion beam from ZEISS—for scanning electron microscopy analysis SEM was performed with. This equipment is dedicated to the study of microscopic structures and inorganic and organic surfaces. The images were taken at an acceleration voltage of 5 kV with a working distance of 4.3–4.5 mm. The detector used was the secondary electron detector of the Everhart Thornley type with the Faraday cup in the sample chamber—resulting in micrographs that highlight the morphology and topography of the analyzed surfaces. Also, we employ an active load compensation system (local) with N2 gas used on the surface of the analyzed sample (CC—charge compensation).

##### FTIR Analysis

The FTIR spectra of the composite samples were recorded using a Jasco 4200 spectrometer (Jasco Inc., Tokyo, Japan) coupled with a JASCO PRO 470-H ATR (Attenuated Total Reflectance diamond) accessory. The samples were measured directly by placing them on the ATR device’s crystal and pressing with a controlled force. The spectra were recorded over the spectral range of 4000–500 cm^−1^ with a resolution of 4 cm^−1^ and 50 scans per spectrum.

##### Dielectric Tests

The dielectric properties, such as the real (ε′) and imaginary parts (ε″) of relative permittivity, along with the tangent of the dielectric loss angle (tg δ = ε″/ε′), of the PP/CMC composites, were determined by dielectric spectroscopy using a Solartron 1260A dielectric spectrometer (Solartron Analytical, Farnborough, UK). The measurements were recorded using an electric field of AC voltage amplitude of 3 V over a frequency range of 1–0.5 MHz and a measuring electrode with a diameter of 30 mm. Samples should be characterized initially and after submission to the above degradation factors by interpreting variation in dielectric characteristics.

##### Influence of Water Exposure on Electrical Properties

To evaluate how water exposure affects electrical insulation in composite materials, a water absorption test was conducted. Three disk-shaped specimens from each material were submerged in water for 10 days. After removal, excess surface water was wiped off, and dielectric characterization was performed at the following five time points: before water exposure (0 h), after immersion (240 h), and then at 480 h, 720 h, and 960 h to track changes over time.

##### Determination of Water Absorption

To determine the water absorption, the tests were carried out according to ISO 62:2008 (en) point 6.4 method 2 [41] on 3 samples from each composite material recipe, and the test cycles were 24, 48, 72, 96, 120, 144, 168, 192, and 216 h. According to the working method [42], the first stage consists of bringing to constant mass. It consists of the initial drying of all samples/samples of composite materials obtained in an oven at 50.0 ± 2.0 °C for a minimum of 24 h. After extracting from the oven, the samples were left to cool, at room temperature, in a desiccator, after which they were weighed. This process was repeated until the mass of the samples was constant with ± 0.1 mg. Samples were chosen from the composite materials that had a mass between 1.1191 and 1.2120 g. These samples, thus prepared, were submerged, each separately, in a plastic vial with a tight lid filled with distilled water. After each cycle, the samples were removed from the water, wiped with a dry cloth, and immediately weighed. The absorbed water content was measured by reimmersing the test samples and weighing them after drying and wiping off excess water after each test cycle. To calculate the amount of water absorbed (in %), the following formula was used:

(1) $$c=\frac{m_{2-}m_{1}}{m_{1}}\times 100\%\;și\;c=\frac{m_{2-}m_{3}}{m_{1}}\times 100\%$$

where:

c—the amount of water absorbed (%);
m_1_—sample mass (mg) dry, before immersion;
m_2_—sample mass (mg) after immersion;
m_3_—mass of the sample (mg) after each cycle of 24 h of immersion in water

##### Determination of Resistance to the Action of UV Radiation

The UV irradiation was performed with a Vilber Lourmat UV lamp at 230 V, 50/60 Hz, with a power of 80 W for 72 h, 144 h, 216 h, and 288 h. After each period of time, dielectric measurements were performed.

##### Determination of Resistance to the Temperature

Determination of resistance to the temperature was achieved with universal oven type Memmert with forced convection model UF30—for determining resistance to temperature action. The samples were subjected to a temperature of 100 °C for 120 h, 240 h, 360 h, and 480 h. After each 120-h cycle, the samples were removed from the oven, placed in a desiccator, and allowed to cool, after which their dielectric characteristics were tested. The test temperatures have been chosen in such a way as to exceed the temperature at which the materials would potentially operate, i.e., inside vehicles (max. 80 °C).

#### 2.2.3. Statistical Analysis

The analyses were performed in triplicates, and the results were presented as the means of three independent experiments. The statistical significance was analyzed by Student’s *t*-test. A value of *p* less than 0.05 was considered significant.

## 3. Results and Discussion

### 3.1. Structural Analysis—SEM (Scanning Electron Microscopy) Analysis

**Figure 2:**
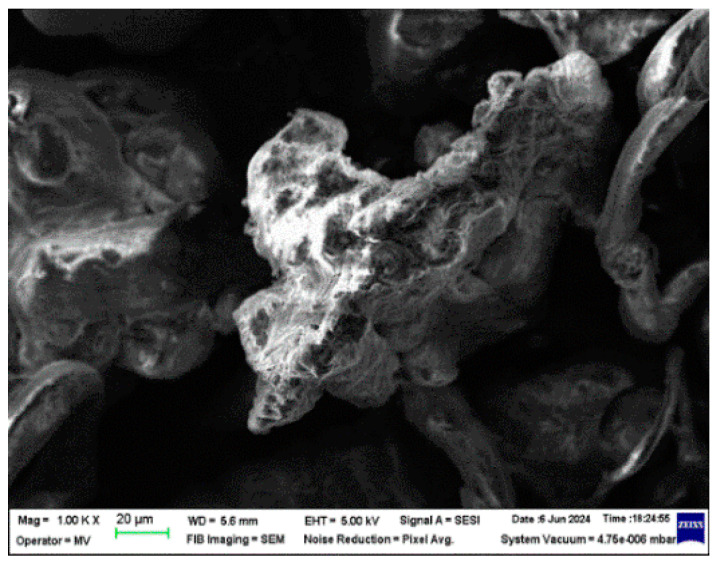
Micrographs for CMC at magnifications 1000×.

**Figure 3:**
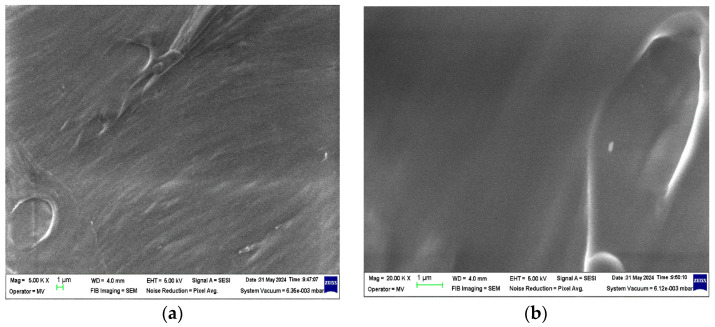
Micrographs for PPr at magnifications (**a**) 5000× and (**b**) 20,000×.

**Figure 4:**
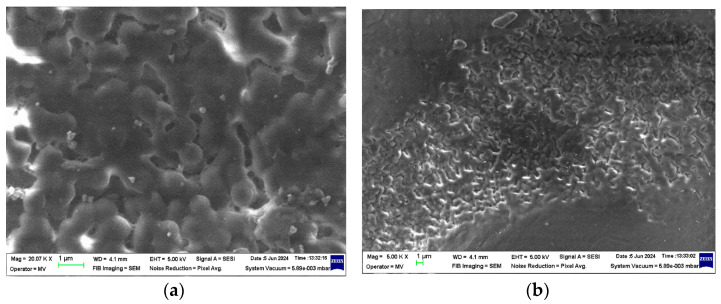
Micrographs for PPr + 5% CMC at magnifications (**a**) 5000× and (**b**) 20,000×.

**Figure 5:**
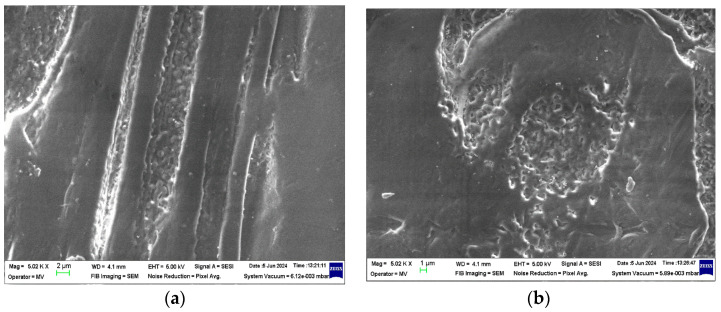
Micrographs for PPr + 10% CMC at magnifications (**a**) 5000× and (**b**) 20,000×.

The structural analyses performed on these samples are presented in Figure 2, Figure 3, Figure 4 and Figure 5.

From the analysis of the structural behavior of these composite materials, it can be observed that for the PPr + 5% CMC composite, the pronounced melting during the composite production process resulted in a polymer filler morphology with a circular shape, indicating a certain level of uniformity and interaction between the polymer and filler at this concentration. The PPr + 10% CMC composite exhibited a greater dispersion of the spherical filler material compared to the 5% CMC composite, suggesting that a higher filler concentration leads to improved distribution within the polymer matrix.

### 3.2. FTIR Analysis

**Figure 6:**
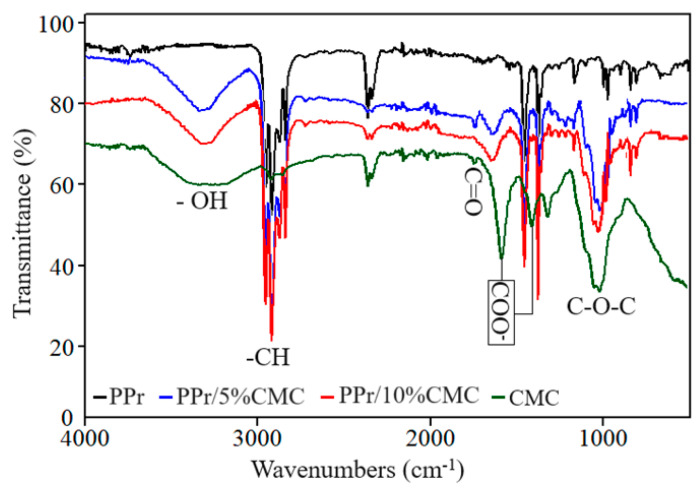
FTIR spectra recorded on PPr-/CMC-based composites.

The FTIR spectra recorded on the CMC sample (Figure 6) contain characteristic bands of CMC as follows [43,44,45,46,47]:

- A broad band between 3600 and 3200 cm^−1^ due to the stretching vibrations of the hydroxyl groups (–OH) in cellulose and carboxylic acids.
- A band between 2950 and 2800 cm^−1^ due to the stretching vibrations of the C-H (e.g., methylene and methine) bonds in the glucose units of cellulose.
- Low-intensity band at 1749 cm^−1^ due to carbonyl (C=O) stretching [48].
- A band between 1591 and 1420 cm^−1^ assigned to the symmetric and asymmetric stretching vibrations of the carboxylate groups (COO–). They are some of the most characteristic peaks for CMC and are often used to identify its presence in a sample.
- A band between 1395 and 1295 cm^−1^ due to in-plane bending vibrations of the C–H bonds.
- A band between 1177 and 921 cm^−1^ attributed to the stretching vibrations within the C-O-C linkages between the glucose units in the cellulose backbone.

The FTIR spectra of polymeric composites primarily exhibit bands characteristic of polypropylene-based materials [47]. These include symmetric and asymmetric stretching vibrations of the methyl group (-CH_3_) at 2952 cm^−1^, 2872 cm^−1^, and 1372 cm^−1^, stretching vibrations of CH_2_ groups at 2921 cm^−1^ and 2852 cm^−1^, and the stretching vibration of –CH at 1457 cm^−1^.

The incorporation of carboxymethyl cellulose into polypropylene is confirmed by the detection of characteristic CMC bands, such as those for –OH, C=O, and C–O–C groups, within the PP FTIR spectrum. Additionally, the observed shift of the carboxylate (COO–) band to higher wavenumbers, from 1587 cm^−1^ in CMC to approximately 1630 cm^−1^ in the PP/CMC composites, suggests an interaction between the PP matrix and CMC, possibly mediated by hydrogen bonding as indicated by the splitting of the OH band [48].

### 3.3. Variation of Dielectric Characteristics

#### 3.3.1. Initial Dielectric Characteristics

**Figure 7:**
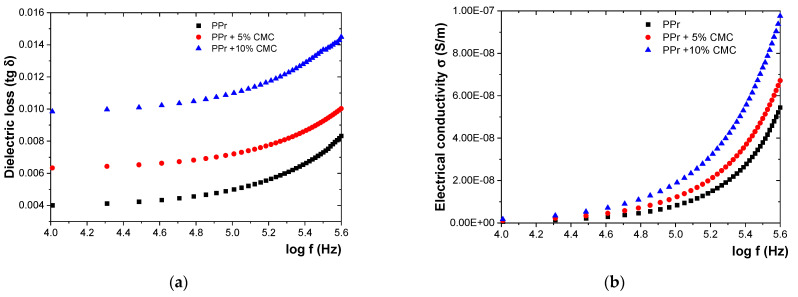
Variation of (**a**) tg δ and (**b**) σ with frequency for initial samples.

Figure 7 shows the variation of initial dielectric characteristics (tangent of dielectric loss angle—tg δ and electrical conductivity—σ) with frequency variation, and Table 3 presents the dielectric characteristics of the composites at frequencies ranging from 0.1 MHz to 0.5 MHz.

In Table 3, the dielectric characteristics of the composites were presented at frequencies between 0.1 MHz and 0.5 MHz.

From Figure 7, it is observed that increasing the percentage of CMC in the polypropylene leads to a significant rise in both the loss tangent (tg δ) and the conductivity (σ) of the composites. Specifically, for the PPr + 5% CMC composite, tg δ and σ increase by approximately 29.23% and 26.68%, respectively. For the PPr + 10% CMC composite, these increases are even more pronounced, with tg δ and σ rising by approximately 90.7% and 85.4%, respectively.

#### 3.3.2. Influence of Water Immersion on Dielectric Properties

Table 4 shows the variations in % of the values of the dielectric characteristics related to the PP base polymer for samples subjected to the action water.

From these variations, it can be said that the most resistant to water action is PPr, and the most affected by water is PPr + 10% CMC. The degradation of composites begins after 480 h of being immersed in water, when a sudden increase in conductivity occurs. This increase in electrical conductivity can be explained by the reaction of OH groups in cellulose, which form hydrogen bonds with water, creating a film of water on the surface of the sample. At the same time, water enters all cracks resulting from composite technology and creates conductive channels. This behavior is justified by the polarizations existing in CMC due to the polar groups it contains. It is found that after 480 h of immersion in water, samples begin to react with water, and in this way, it can be said that their dielectric properties begin to deteriorate.

##### Determination of Water Absorption

Thus, the amount of water absorbed by the PPr/CMC type composite materials was analyzed by comparison with the control, i.e., polypropylene (PPr). The experimental results are presented in Table 5.

The obtained experimental results confirm that pure polypropylene (PPr) reaches saturation after approximately 120 h of immersion in water. In contrast, the PPr + 5% CMC composite begins to show signs of saturation around 168 h. For the PPr + 10% CMC composite, saturation has not been reached, and it continues to absorb water even after 216 h of immersion.

#### 3.3.3. Variation of Dielectric Characteristics with Temperature

**Figure 8:**
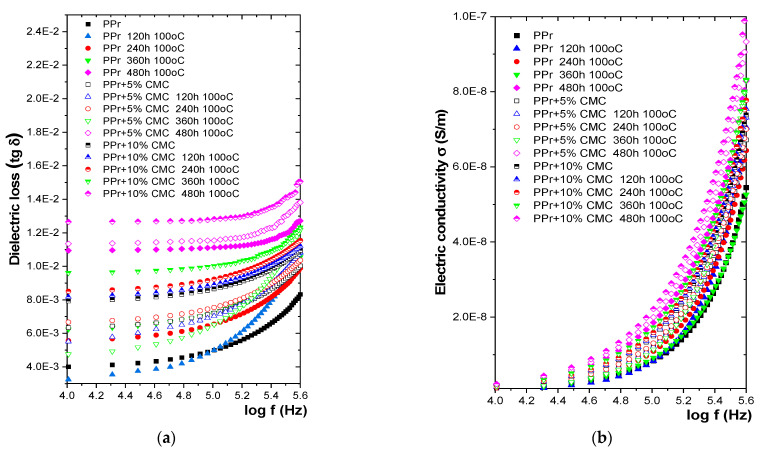
Variation of (**a**) tg δ and (**b**) σ with frequency at temperature action.

Figure 8 shows the variation of electrical characteristics with frequency for samples subjected to a temperature of 100 °C for 120, 240 h, 360 h, and 480 h. An increase in electrical conductivity is observed for all composites studied after 480 h of exposure to temperature. Table 6 shows the variations in % of the values of the dielectric characteristics related to the PPr base polymer for samples subjected to the action of temperature.

From the experimental results obtained, it is observed that even after 360 h at 100 °C there is no substantial degradation/increase in electrical conductivity, which can be explained by the fact that the polymer matrix made of PPr is very resistant to temperature action. After 240 h of temperature submission, it is found that in the case of PP there is a slight decrease in electrical conductivity, which indicates the start of a cross-linking process, which continues up to 360 h, ending at 480 h. From these variations, it can be said that the most resistant to temperature action is PPr, and the most affected by temperature action is also PPr + 10% CMC. The degradation of composites under the action of temperature begins slowly, respectively, after 480 h.

#### 3.3.4. Variation of Dielectric Characteristics with UV Radiation

**Figure 9:**
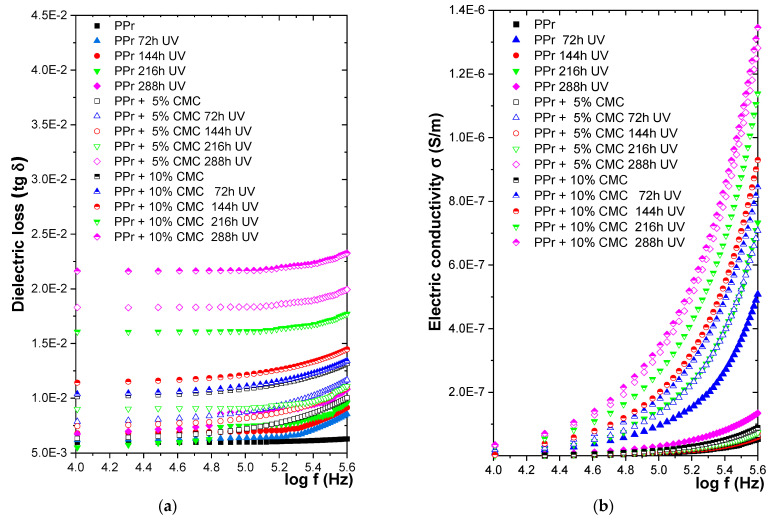
Variation of (**a**) tg δ and (**b**) σ with frequency with UV radiation.

Figure 9 presents the variation of electrical characteristics with frequency for samples subjected to UV radiation for four cycles of 72 h, 144 h, 216 h, and 288 h. It is found that after maintenance under the action of UV radiation, the variations in dielectric characteristics related to the blank PP are at:

Table 7 shows the variations in % of the values of the dielectric characteristics related to the PP base polymer for samples subjected to the action of UV radiation.

A sharp increase in electrical conductivity is observed for all studied composites after the first 72 h of UV radiation. Generally, the UV radiation energy disrupts the strong bonds holding the polymer chains together, inducing scissions and creating highly reactive loose ends called free radicals. These free radicals set off a chain reaction of further breakage with neighboring chains, ultimately weakening and degrading the entire polymer structure. Polypropylene is known to be highly susceptible to degradation by UV radiation because of the small amount of vinyl, vinylidene groups, and catalyst residues, which are playing a photo-initiator role. In the case of UV radiation, it is also found that the PP + 10% CMC composite suffers the strongest degradation, the degradation highlighted by the sharp increase in electrical conductivity.

## 4. Conclusions

In this work, PPr-/CMC-type composites were obtained from recycled raw materials. The purpose of obtaining these composites is to identify one that, under the influence of climatic factors (temperature, UV radiation, and humidity), will destroy the polymer chains as quickly as possible. The composites were obtained by extrusion and melt injection—the classic methods of obtaining polymer composites. Structural analyses were carried out on these composites, which identified the fact that in the PPr + 5% CMC type composite, a more pronounced melting occurs in the process of obtaining it, and a polymer morphology + the filler in a circular shape was highlighted, and in the PPr composite + 10% CMC, it is found that the spherical CMC has a greater dispersion than the one with 5% CMC. The FTIR spectra of the composites show bands characteristic of polypropylene. The presence of CMC in PP is confirmed by the detection of characteristic CMC bands. Following the tests to determine the resistance to the action of humidity, it is found that the electrical conductivity of the PP + 10% CMC composite increases the most compared to PP, which leads us to the conclusion that after 480 h of immersion in water, the PPr + 10% composite CMC begins to degrade. Tests to determine resistance to UV radiation indicate a substantial increase in electrical conductivity after the first cycle of UV radiation. Following exposure to UV radiation, the PPr + 10% CMC composite suffers the strongest degradation. Following the tests to determine the temperature resistance, it is found that the most resistant to the action of temperature is PP, and the most affected by the action of temperature is also PPr + 10% CMC. The degradation of the composites under the influence of temperature begins slowly, respectively, after 480 h.

In the context of the circular economy and increasing the recyclability of cars towards the limit imposed by “fully recyclable cars”, following this research, we can recommend the PPr + 10% CMC composite as a recyclable composite that degrades more easily in atmospheric conditions than polypropylene.

## Figures and Tables

**Table 1:** Heating zones.

**Heating zones of the extruder screw**	**6**	**5**	**4**	**3**	**2**	**1**
**Temperature (°C)**	240	230	220	210	200	190
**Heating zones of the injection machine screw**		5	4	3	2	1
**Temperature (°C)**		230	215	200	185	170

**Table 2:** Concentrations and coding of composites.

Composite Concentration (wt.%) PPr/CMC	Coding
0/100	CMC
100/0	PPr + 0% CMC
95/5	PPr + 5% CMC
90/10	PPr + 10% CMC

**Table 3:** Dielectric characteristics of composites at frequencies between 0.1 MHz and 0.5 MHz.

Frequency (MHz)		Electrical Characteristics	0.1	0.2	0.3	0.4	0.5
	Materials						
PPr		tg δ	8.34 × 10^−3^	7.15 × 10^−3^	6.36 × 10^−3^	5.96 × 10^−3^	5.14 × 10^−3^
PPr		σ	1.40 × 10^−8^	2.40 × 10^−8^	3.20 × 10^−8^	4.00 × 10^−8^	4.22 × 10^−8^
PPr + 5% CMC		tg δ	1.02 × 10^−2^	8.79 × 10^−3^	8.31 × 10^−3^	7.88 × 10^−3^	6.85 × 10^−3^
PPr + 5% CMC		σ	1.76 × 10^−8^	3.02 × 10^−8^	4.28 × 10^−8^	5.41 × 10^−8^	5.76 × 10^−8^
PPr + 10% CMC		tg δ	1.33 × 10^−2^	1.23 × 10^−2^	1.15 × 10^−2^	1.11 × 10^−2^	1.04 × 10^−2^
PPr + 10% CMC		σ	2.31 × 10^−8^	4.27 × 10^−8^	5.94 × 10^−8^	7.64 × 10^−8^	8.83 × 10^−8^

**Table 4:** The experimental results for the samples subjected to the action of water.

Exposure Time (hours); % Variation		240		480		720		960	
		Approx. % Increase							
	Dielectric Composites Characteristics	tg δ	σ	tg δ	σ	tg δ	σ	tg δ	σ
PPr		5.87%	3.51	9.33	863.08	17.6	7.74	23.41	20.04
PPr + 5% CMC		75.83	64.78	105.06	1666.52	115.60	99.56	148.76	137.39
PPr + 10% CMC		192.72	167.75	217.07	2564.23	370.78	4076.02	452.7	4814.49

**Table 5:** Water absorption for PP/CMC composite materials.

Samples	c 24 h	c 48 h	c 72 h	c 96 h	c 120 h	c 144 h	c 168 h	c 192 h	c 216 h
PPr 1	0.01	0.02	0.02	0.02	0.03	0.033	0.04	0.04	0.05
PPr 2	0.02	0.03	0.04	0.09	0.13	0.18	0.20	0.20	0.21
PPr 3	0.03	0.05	0.10	0.18	0.24	0.29	0.29	0.30	0.30
variation from the initial mass (%)		34.76	63.31	79.14	84.90	87.90	88.44	88.67	89.16
PPr + 5% CMC 1	0.04	0.07	0.07	0.08	0.10	0.14	0.25	0.26	0.26
PPr + 5% CMC 2	0.03	0.02	0.06	0.08	0.14	0.29	0.32	0.33	0.34
PPr + 5% CMC 3	0.04	0.05	0.08	0.13	0.17	0.19	0.25	0.27	0.28
variation from the initial mass (%)		13.35	43.86	58.57	70.58	80.89	85.35	86.07	86.34
PPr + 10% CMC 1	0.05	0.07	0.08	0.12	0.18	0.29	0.43	0.23	0.35
PPr + 10% CMC 2	0.05	0.06	0.07	0.09	0.15	0.21	0.22	0.33	0.37
PPr + 10% CMC 3	0.04	0.06	0.08	0.10	0.12	0.17	0.25	0.42	0.44
variation from the initial mass (%)		26.09	41.42	55.27	68.50	78.74	84.40	85.62	87.82

**Table 6:** The experimental results for the samples subjected to the action of 100 °C temperature.

Exposure Time (hours); % Variation		120		240		360		480	
		Approx. % Increase							
	Dielectric Composites Characteristics	tg δ	σ	tg δ	σ	tg δ	σ	tg δ	σ
PPr		19.29	20.13	23.24	4.23	33.2	1.62	77.62	77.62
PPr + 5% CMC		33.85	35.64	34.19	32.39	40.74	40.74	88.91	79.72
PPr + 10% CMC		35.64	33.85	55.52	49.35	66.35	59.63	107.30	96.61

**Table 7:** The experimental results for the samples subjected to the action of UV radiation.

Exposure Time (hours); % Variation		72		144		216		288	
		Approx. % Increase (i) % Decrease (d)							
	Dielectric Characteristics Composites	tg δ	σ	tg δ	σ	tg δ	σ	tg δ	σ
PPr		13.4 (i)	881.56 (i)	38.83 (d)	26.27 (d)	16 (d)	1251.35 (d)	16 (d)	1251.35 (d)
PPr + 5% CMC		43.32 (i)	1251.07 (i)	35.36 (d)	38.42 (i)	40.5 (d)	37.92 (d)	40.5 (d)	37.92 (d)
PPr + 10% CMC		71.44 (i)	1493.02 (i)	86.14 (i)	1657.07 (i)	37.92 (i)	2076.69 (i)	135.94 (i)	2076.69 (d)

## Data Availability

Data are contained within the article.
